# Analysis of the factors influencing reoperation for severe finger scar contracture in children—clinical data analysis of 61 children

**DOI:** 10.3389/fped.2026.1850509

**Published:** 2026-06-25

**Authors:** Weiwei Qi, Yinqiu Li, Chen Chen, Yuan Cheng

**Affiliations:** Department of Burn and Plastic Surgery, Anhui Provincial Children's Hospital, Hefei, China

**Keywords:** children, fingers, joint contracture, SCAR, skin graft

## Abstract

**Objective:**

To explore the factors influencing unplanned reoperation after surgery for severe finger scar contracture in children.

**Methods:**

The clinical data of children who underwent skin grafting for severe finger scar contracture at the burn and plastic surgery department of Anhui children’s hospital from May 2015 to June 2019 were collected, including: etiology, age at surgery, defect area, presence of wound infection, and involvement of multiple joints. We divided the patients into the non-reoperation group (Group A) and the reoperation group (Group B).

**Results:**

A total of 61 children were collected, including 42 boys and 19 girls, with a total of 93 fingers. Among them, 43 children had no repeat surgery, and 18 children had repeat surgery. The finger defect area in Group A was 1.31 ± 0.37 cm^2^, while in Group B, it was 2.24 ± 0.60 cm^2^. The time from wound healing in Group A was 8.43 ± 3.11 months, while in Group B, it was 13.67 ± 2.74 months. The number of joints crossed by the scar in Group A was 1.09 ± 0.29, and in Group B, it was 1.57 ± 0.68. There were significant differences between the two groups (*p* < 0.05). In multivariate logistic regression, the results showed that defect area (OR = 17.64, 95% CI: 2.36−131.81, *P* = 0.005) and time since the first surgery (OR = 1.39, 95% CI: 1.03-1.91, *P* = 0.03) independently predicted the need for repeat surgery.

**Conclusion:**

The area of skin defect and the time from wound healing in finger scar contractures are independent factors influencing the need for reoperation.

## 1 Introduction

Due to children's innate curiosity and poor self-protection awareness, they are highly susceptible to hand injuries. Among children, although the hands account for only about 4% of the total body surface area, 35% of burn injuries in children under the age of 5 occur on the hands (1, 2). For these children, who are in the growth and development stages, the most serious consequence of hand thermal and traumatic injuries is the scar contracture of the joints in the hand after wound healing. which not only requires a long recovery period and multiple surgeries but also affects the psychological health of school-age children (3). Multiple studies have shown that for children with hand injuries, the post-injury treatment, the timing of surgery, and postoperative rehabilitation are crucial for improving hand function prognosis (4, 5). Research on subsequent treatment for children who develop scar contractures remains limited, and clinical experience with children undergoing multiple surgeries has not yet been systematically summarized. Therefore, this study analyzes clinical data from children with severe finger scar contractures and explores the factors influencing unplanned reoperation.

## 2 Materials and methods

### 2.1 General data

Clinical data were collected from children who underwent full-thickness skin grafting for severe finger scar contracture at the Department of Burn and Plastic Surgery, Anhui Children's Hospital, from May 2015 to June 2019. The collected variables included etiology, age at surgery, defect area, wound infection, and involvement of multiple joints. The patients were divided into two groups: the non-reoperation group (Group A) and the reoperation group (Group B). In this study, reoperation was defined as an unplanned second or subsequent operation performed after the initial scar release and full-thickness skin grafting because of recurrent scar contracture, progressive deformity, or functional limitation during follow-up. Planned staged procedures determined at the time of the initial operation were not considered reoperations. This study was approved by the Anhui Provincial Children's Hospital Training Program for Outstanding Residents (No. eyrc035), and informed consent was obtained from all patients and their families.

### 2.2 Inclusion and exclusion criteria

According to the Schneider severity classification, patients were categorized into mild, moderate, and severe degrees. Inclusion criteria: 1) Age <16 years; 2) Finger scar contracture classified as severe based on Schneider's severity scale; 3) Full-thickness skin grafting surgery performed. Exclusion criteria: 1) Age >16 years; 2) Family history of keloid formation; 3) Scar contracture involving joints other than fingers; 4) Failure to adhere to standardized post-surgery anti-scar treatment and rehabilitation training.

### 2.3 Finger ROM (range of motion) schneider severity classification

The range of motion of the affected finger joints was measured using a standard finger goniometer, and the range of motion of the involved joints was recorded. The severity of scar contracture of the affected fingers was then classified as mild, moderate, or severe according to the Schneider severity grading criteria. For children with involvement of multiple joints, the joint with the most severe limitation of motion was used as the basis for the final severity classification (6) (see Table 1).

**Table 1:** Range of motion evaluation form.

Scar Contracture Joints	Movement	Mild	Moderate	Severe
Wrist	Flexion	41–59	21–40	0–20
	Extension	41–59	21–40	0–20
	Radial Deviation	13–19	7–12	0–6
	Ulnar Deviation	20–29	10–19	0–9
Metacarpophalangeal joints 2–5	Flexion	60–89	30–59	0–29
	Extension	−1–30	−31–60	−61–90
Proximal interphalangeal joints 2–5	Flexion	67–99	34–66	0–33
	Extension	−1–33	−34–66	−67–100
Distal interphalangeal joints 2–5	Flexion	47–69	24–46	0–23
	Extension	−1–23	−24–46	−47–70
Thumb	Flexion	48–69	24–47	0–23
Webbing of fingers	Extension	−1–23	−24–46	−47–70

### 2.4 Postoperative anti-scar treatment

Postoperatively, the affected finger was immediately immobilized with an orthosis to maintain the corrected position. After stable wound healing was achieved, silicone gel was applied twice daily, in the morning and evening. Following stable graft take or removal of the Kirschner wire, active and passive range-of-motion exercises, scar massage, and grasping training were initiated. During the later rehabilitation period, nighttime splinting was continued for 3–6 months, with regular follow-up and adjustment as needed.

### 2.5 Follow-up

After discharge, the patients are followed up every two weeks for the first month, and then monthly thereafter. The median follow-up time was 6.5 years.

### 2.6 Statistical methods

SPSS 22.0 was used for statistical analysis of clinical data. Measurement data were described as mean ± standard deviation or median and interquartile range. Comparisons between the two groups were made using independent sample t-tests (for normal distribution and equal variances) or Wilcoxon rank-sum tests (for non-normal distribution or unequal variances). For comparison of categorical data, the Chi-square test was used. Fisher's exact probability method was applied when the sample size in a 2 × 2 table was less than 40 or if at least one cell had a frequency of T < 1. Binary logistic regression was used to analyze influencing factors. A *p*-value < 0.05 was considered statistically significant.

## 3 Results

### 3.1 General clinical data

**Figure 1:**
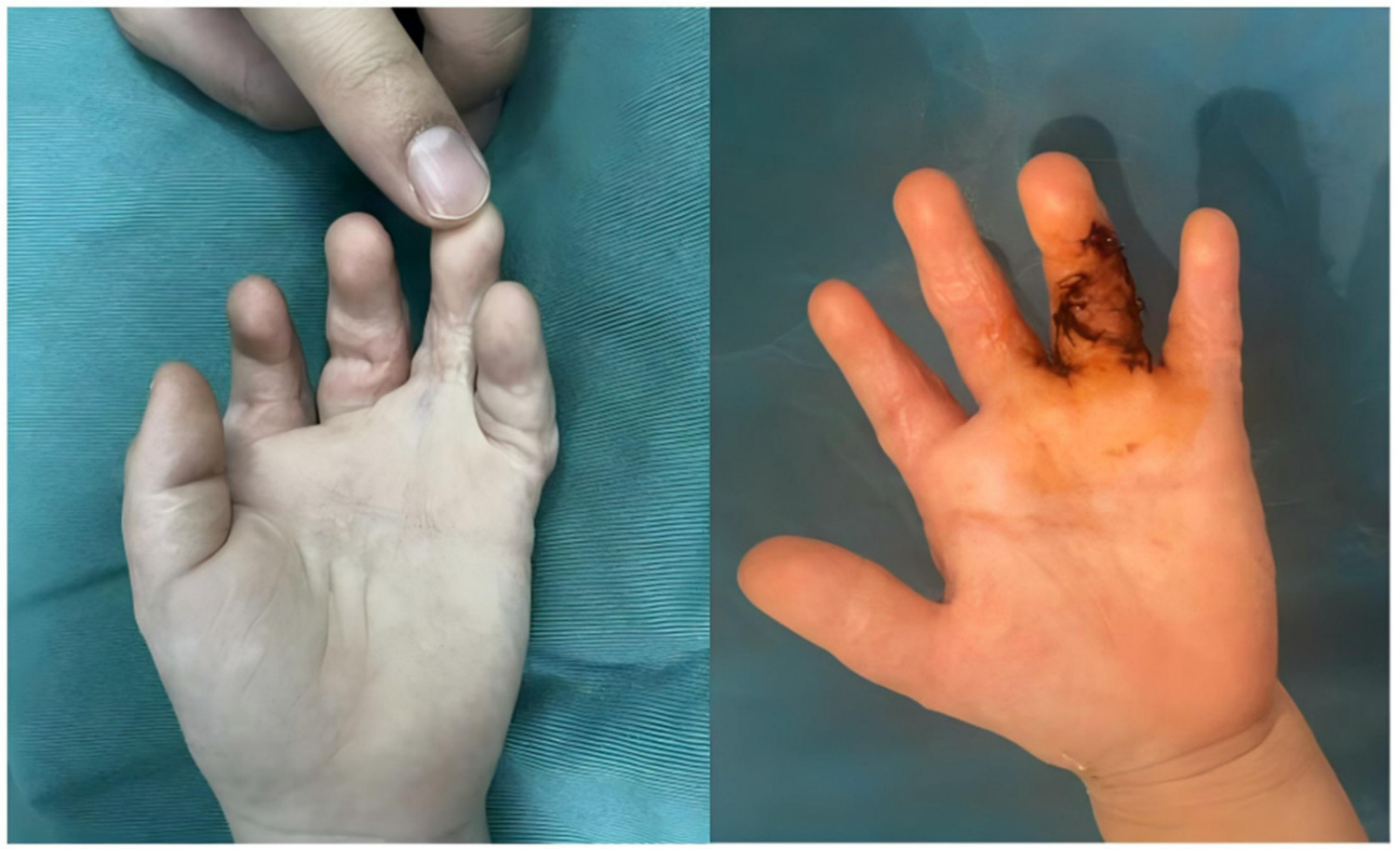
(Group A)A 2-year-and-10-month-old boy presented with volar scar contracture of the ring finger, resulting in flexion deformity and limited extension. After scar release and full-thickness skin grafting,this case showed a relatively smaller defect area and more limited finger/joint involvement.

**Figure 2:**
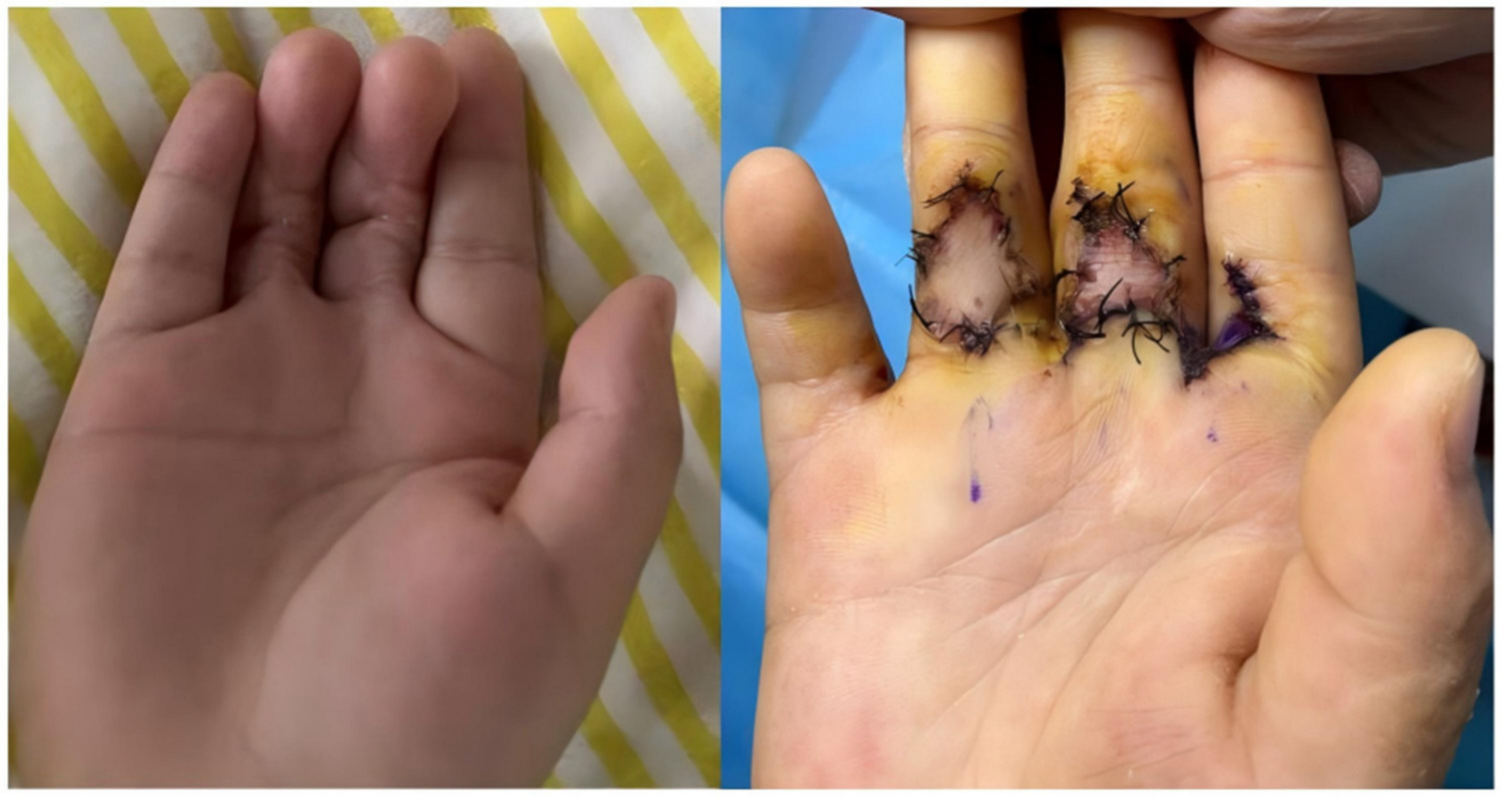
(Group B) A 1-year-and-7-month-old boy presented with volar scar contracture of the middle and ring fingers, resulting in flexion deformity and limited extension. After scar release and full-thickness skin grafting, the volar defects of the involved fingers were covered with skin grafts. This case showed a relatively large defect area and multiple-finger involvement.

Among the 61 children included in this study, 42 were boys and 19 were girls, with a total of 93 affected fingers. Of these, 43 children with 72 affected fingers were assigned to the non-reoperation group (Group A), and 18 children with 21 affected fingers were assigned to the reoperation group (Group B), Table 2. Representative cases from the two groups are shown in Figure 1 and Figure 2. There were no statistical differences between the two groups in terms of age and gender. The finger defect area in Group A was 1.31 ± 0.37 cm^2^, while in Group B, it was 2.24 ± 0.60 cm^2^. The time from wound healing in Group A was 8.43 ± 3.11 months, while in Group B, it was 13.67 ± 2.74 months. The number of joints crossed by the scar in Group A was 1.09 ± 0.29, and in Group B, it was 1.57 ± 0.68. There were significant differences between the two groups (*p* < 0.05) Table 3.

**Table 2:** General clinical data.

*N* = 61		Non-reoperation Group (Group A)	Reoperation Group (Group B)	χ^2^/Z/T value	*P* value
Age at first surgery (months)		37.75 ± 26.19	33.71 ± 23.53	0.62	0.54
Gender	Male	30	12	0.06	0.81
	Female	13	6		
Cause of Injury	Thermal Injury	36	15	−0.04	0.97
	Trauma	5	3		
	Other	2	0		

**Table 3:** Comparison of clinical data between the Two groups.

*N* = 93		Non-reoperation Group (Group A)	Reoperation Group (Group B)	χ^2^/Z/T value	*P* value
Defect Area (cm2)	1.53 ± 0.76	2.13 ± 0.66	−8.57	<0.001	
Time from Wound Healing to First Surgery (months)	7.02 ± 4.41	14.33 ± 12.43	−6.96	<0.001	
Number of Joints Crossed (count)	1.16 ± 0.37	1.50 ± 0.65	−0.31	<0.001	
Primary Wound Healing Time (days)	23.29 ± 3.14	22.05 ± 5.03	−1.76	0.08	
Wound Healing Method	Spontaneous Healing	68	15	/	0.12
	Surgical Intervention	4	6		
Multiple Fingers are Affected	Yes	25	7	0.01	0.91
	No	47	14		
Postoperative Infection	Yes	2	1	/	0.65
	No	70	20		
Adhesive syndactyly	Yes	4	3	/	0.19
	No	68	18		
Surgical Method	Full-thickness Skin Graft	67	17	/	0.11
	Full-thickness Skin Graft + Adjacent Flap	5	4		
postoperative Kirschner wire fixation	Yes	21	8	0.60	0.44
	No	51	13		
Finger	Thumb	3	2	−0.49	0.63
	Index Finger	10	3		
	Middle Finger	26	8		
	Ring Finger	23	4		
	Little Finger	10	4		
Whether the Dominant Hand	Yes	52	14	0.24	0.62
	No	20	7		

### 3.2 Multivariate logistic regression analysis

**Figure 3:**
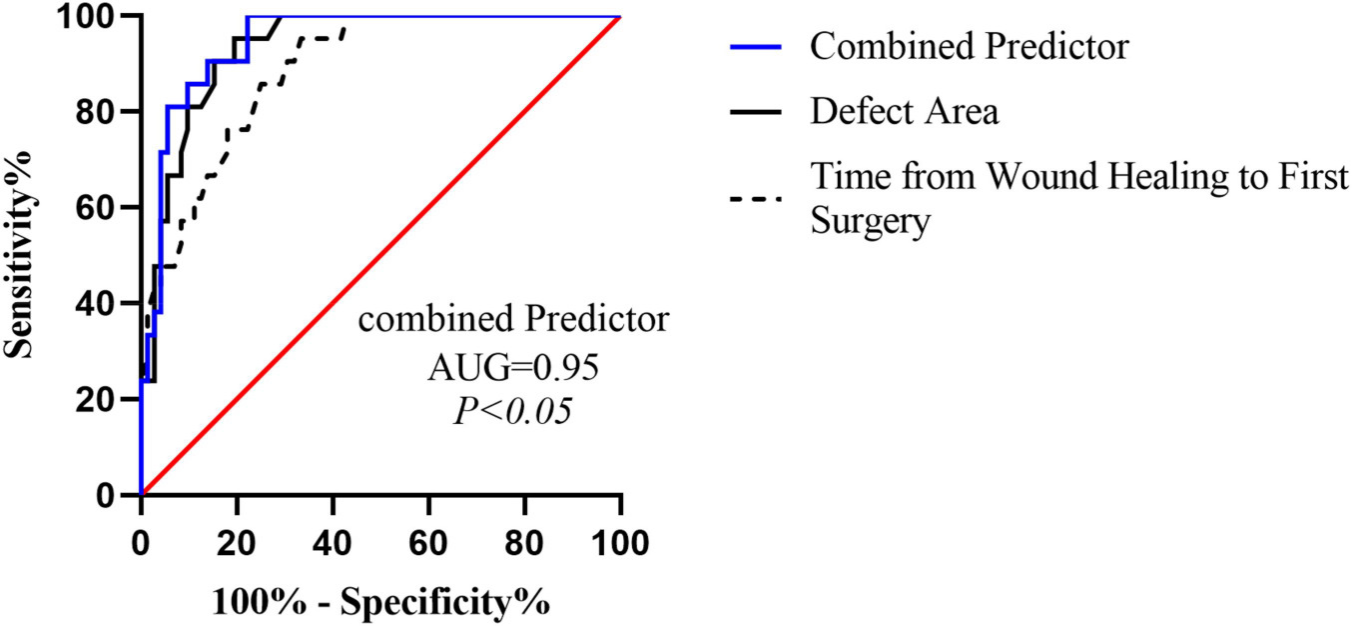
ROC curve.

Factors with statistical differences between the two groups—defect area, time from the first surgery, and the number of joints crossed—were included in the multivariate logistic regression. The results showed that defect area (OR = 17.64, 95% CI: 2.36-131.81, *P* = 0.005) and time from the first surgery (OR = 1.39, 95% CI: 1.03-1.91, *P* = 0.03) were independent factors influencing the need for reoperation. As shown in Table 3, in the ROC curve of the prediction model, the AUC for defect area was 0.93 (*P* < 0.05), with an optimal cutoff value of 1.5 cm^2^, sensitivity of 95.2%, and specificity of 73.6%. The AUC for time from the first surgery was 0.89 (*P* < 0.05), with an optimal cutoff value of 9 months, sensitivity of 95.2%, and specificity of 58.3%. The AUC for the combined prediction was 0.95 (*P* < 0.05) Table 4, Figure 3.

**Table 4:** Multivariate logistic regression analysis.

Factors	B	SE	Walds	OR Value	*P* Value	95% CI
Defect Area After Scar Release (cm^2^)	2.87	1.03	7.82	17.64	0.005	2.36–131.81
Time from Wound Healing to First Surgery (months)	0.34	0.16	4.51	1.39	0.034	1.03–1.91
Number of Joints Crossed (count)	0.22	0.88	0.60	1.24	0.81	0.22–6.96

*P* < 0.05 indicates that the difference is statistically significant.

## 4 Discussion

Currently, the severity classification of hand scar contracture is not fully standardized. It is generally believed that normal ROM is divided into three parts to determine mild, moderate, and severe contracture (7). For patients with less than 50% of normal ROM, flap or skin graft surgery is usually recommended (8). In pediatric patients, those with less than 50% of normal ROM, after the first surgery, may require reoperation or multiple surgeries due to the rapid growth phase of children. This difference in the potential growth capacity of free skin grafts compared to the surrounding normal skin and tissue can lead to the need for further surgeries. Currently, there is no globally unified standardized guideline for the treatment of pediatric finger scar contracture. Most research focuses on treatment modality selection, surgical approach, and postoperative management (9, 10). In clinical practice, it is difficult to assess whether a child requires reoperation or how many surgeries are necessary. The main aim of this study is to preliminarily explore the independent predictive factors for reoperation after surgery for pediatric finger scar contracture, providing a basis for early identification of high-risk children, optimizing surgical strategies, and postoperative management.

### 4.1 The impact of skin defect area on recurrence after scar contracture surgery

For scar contracture, full-thickness skin grafting is generally recommended to minimize secondary contraction. However, in a study on pediatric scars, the recurrence rate in the full-thickness skin graft group ranged from 0% to 11%, while in another study on pediatric palm contractures, the recurrence rate for children receiving full-thickness skin grafts was 9.4% (11, 12). In our study, the recurrence rate was 22.58%, significantly higher than that reported in previous studies. In our study, the finger defect area in Group A was 1.31 ± 0.37 cm^2^, while in Group B, it was 2.24 ± 0.60 cm^2^. There was a significant difference between the two groups (*p* < 0.05). In the multivariate logistic regression, the defect area (OR = 17.64, 95% CI: 2.36-131.81, *P* = 0.005) was significant. the AUC for defect area was 0.93 (*P* < 0.05), with an optimal cutoff value of 1.5 cm^2^, sensitivity of 95.2%, and specificity of 73.6%. Defect area is an independent risk factor for reoperation after finger scar contracture surgery. This is consistent with Zhang et al.'s research, which suggests that the area of transplanted skin is an independent factor influencing surgical complications (13). The finger scar contracture's skin defect area is an important determinant for the choice of surgical technique. For example, in early research by Sheridan et al., scars with skin defects <2 cm^2^ were treated with local flaps, and medium defects were treated with perforator flaps (14). Cho et al. developed a dimensional classification system for hand scars, determining the surgical approach based on the size of the scar defect, the availability of healthy skin around the defect, and the blood circulation in the defect bed (15). Additionally, although full-thickness grafts have the least secondary contraction, Barry et al.'s mouse study showed that all full-thickness skin grafts initially contract (over two to three weeks), with body skin grafts contracting the most before expanding to 65% of their original area. Therefore, the larger the defect area of hand scars, the greater the potential for contraction after full-thickness skin grafting, leading to recurrence of scar contracture.

### 4.2 The impact of time from wound healing to first surgery on recurrence after scar contracture surgery

We identified the time from wound healing to the first surgery as another independent risk factor. In our study, the time from wound healing in Group A was 8.43 ± 3.11 months, while in Group B, it was 13.67 ± 2.74 months. The time from the first surgery (OR = 1.39, 95% CI: 1.03-1.91, *P* = 0.03) showed that the AUC for time from the first surgery was 0.89 (*P* < 0.05), with an optimal cutoff value of 9 months, sensitivity of 95.2%, and specificity of 58.3%. This is consistent with Yang et al.'s research, which found that the longer the interval between injury and scar formation and receiving surgical treatment, the poorer the effectiveness of scar treatment (16). Typically, after skin injury, the process of scar formation follows a relatively predictable timeline. Hypertrophic scars usually begin to appear 1-2 months after the injury, peak at 3-6 months, and then enter a maturation and regression phase that lasts 6 months to 2 years or even longer (17). A systematic review on scar prevention suggests that over time, scar tissue undergoes “maturation” and “strengthening” in both structure and biological behavior, reducing its responsiveness to treatment and making it more resistant. The longer the duration of the condition, the larger the scar tissue becomes and the richer its blood supply (18). In the studies by Yang and Yeong et al., it is believed that performing surgery after the scar enters the stable phase can reduce the occurrence of scar contracture and deformity. However, most patients cannot receive timely medical treatment, which affects the final effectiveness of scar treatment (16, 19). Therefore, we believe that in order to prevent missing the appropriate time for surgery, follow-up visits and post-discharge education for the child's family should be strengthened. This way, any signs of scar contracture in children can be detected and surgery performed in a timely manner.

### 4.3 The impact of the number of joints crossed on recurrence after scar contracture surgery

In the special pediatric population, long-term hand scar contractures cause skin defects on the surfaces of movable joints. During functional reconstruction, attention must also be given to the ratio of soft tissue contracture to bone length. The maturity of burn scars and the expected growth and development in children play a role in choosing the surgical approach that maximizes function and range of motion. In our study, the number of joints crossed by the scar in Group A was 1.09 ± 0.29, and in Group B, it was 1.57 ± 0.68, with a significant difference between the two groups (*p* < 0.05). Although not an independent risk factor, we believe this is an important factor unique to children. In Cho et al.'s research, multi-joint and long-axis involvement in scar contracture was considered severe, and compared to skin grafting, they preferred “flap reconstruction” to better withstand longitudinal tension and stretch over time, thereby reducing the recurrence of scar contracture (15).Furthermore, multi-joint contractures in children often accompany involvement of deep structures such as tendons and joints during growth and development. Simpson et al. aimed to customize surgical plans aimed at restoring maximum function by considering the ratio of soft tissue to bone length (20). They believed that hypertrophy and hardening of burn scars worsen the ratio, and in their study, all patients who achieved a ratio of soft tissue to bone length of 0.8:1 or better recovered optimal joint position and function. Additionally, autologous fat grafting may be a feasible method to improve the effectiveness of treatment for children with multi-joint involvement by improving scar flexibility. Research shows that after grafting, the flexibility of the scar improves, making it softer and more stretchable (21).

## 5 Conclusion

This study, through the analysis of clinical data from 61 children with severe finger scar contracture, found that skin defect area and the time from wound healing to the first surgery are independent risk factors influencing whether the child needs reoperation. The larger the defect area and the longer the time between surgeries, the higher the risk of reoperation. The larger the defect area and the longer the time between surgeries, the higher the risk of reoperation. Additionally, although the number of joints crossed did not become an independent factor in the multivariate analysis, it still showed significant differences in the univariate analysis, suggesting its important clinical significance in the recurrence of pediatric scar contracture.

### Limitations and drawbacks

This study is a single-center retrospective study with a relatively small sample size, especially with fewer cases in the reoperation group, which may affect the stability and generalizability of the statistical results. Additionally, the study did not include potential influencing factors such as postoperative rehabilitation compliance, family socioeconomic background, and nutritional status of the children, which may have a significant impact on scar healing and functional recovery. Furthermore, although the follow-up time was a median value, not all children were uniformly followed up for the long term. Some children may undergo reoperation in the future, leading to potential bias in the results. Future studies should be multi-center, prospective, and involve larger sample sizes to further validate the conclusions of this study, and incorporate more clinical and family environment variables to build a more comprehensive predictive model.

## Data Availability

The original contributions presented in the study are included in the article/Supplementary Material, further inquiries can be directed to the corresponding author.

## References

[1] Battle CE Evans V James K Guy K Whitley J Evans PA. Epidemiology of burns and scalds in children presenting to the emergency department of a regional burns unit: a 7-year retrospective study. Burns Trauma. (2016) 4:19. 10.1186/s41038-016-0047-7 27574688 PMC4964307

[2] Takasi P Purbarar F Tamizi A Ghardashpoor E. High rate of negligence induced burns in children: a rising cause for concern of the world's Burn community. JNRCP. (2024) 2(2):118–20. 10.32598/jnrcp.23.97

[3] Sun Y Wang Y Jiang YJ Sun Y Zhang J. Systematic review of physical and psychosocial problems of burned children aged 5 years and below after discharge. Zhonghua Shao Shang Za Zhi. (2019) 35(5):371–8. 10.3760/cma.j.issn.1009-2587.2019.05.009 31154736

[4] Nolan MM Reppucci ML Urban A Kierulf G Fields T Boulter T. A single Institution's Recent experience with pediatric hand burns. J Burn Care Res. (2023) 44(4):955–62. 10.1093/jbcr/irac174 36394415 PMC10321372

[5] Abbas P Choe M Ridelman E Angst BA Klein JD Shanti CM. 737 Treadmill friction hand injuries in the pediatric patient. J Burn Care Res. (2020) 41(Supple1):S200–1. 10.1093/jbcr/iraa024.320

[6] Schneider JC Holavanahalli R Helm P O'Neil C Goldstein R Kowalske K. Contractures in burn injury part II: investigating joints of the hand. J Burn Care Res. (2008) 29(4):606–13. 10.1097/BCR.0b013e31817db8e1 18535473

[7] Korp K Richard R Hawkins D. Refining the idiom “functional range of motion” related to burn recovery. J Burn Care Res. (2015) 36(3):e136–45. 10.1097/BCR.0000000000000149 25162944

[8] Hudson DA Renshaw A. An algorithm for the release of burn contractures of the extremities. Burns. (2006) 32(6):663–8. 10.1016/j.burns.2006.02.009 16905261

[9] Kumar S Khan FAA Ali H Kiran S. Surgical management of post burn hand deformities. Pak J Med Sci. (2020) 36(6):1387–91. 10.12669/pjms.36.6.2206 32968414 PMC7501031

[10] Terziqi H Sopjani I Gjikolli B Muqaj G Mustafa M. Algorithms for management of post-burn contracture in upper extremity in children. Ann Burns Fire Disasters. (2021) 34(2):192–8. PMID: 34584510 34584510 PMC8396151

[11] Chandrasegaram MD Harvey J. Full-thickness vs split-skin grafting in pediatric hand burns–a 10-year review of 174 cases. J Burn Care Res. (2009) 30(5):867–71. 10.1097/BCR.0b013e3181b48610 19692910

[12] Al-Qattan MM. Campfire burns of the palms in crawling infants in Saudi Arabia: results following release and graft of contractures. J Burn Care Res. (2009) 30(4):616–9. 10.1097/BCR.0b013e3181ac0298 19506489

[13] Zhang M Fang Y Li H Shi S Chen J Tang F. Prognostic analysis of skin scar loosening and tissue-expansive autologous skin grafting in the treatment of skin postburn scars. J Craniofac Surg. (2022) 34(5):e411–5. 10.1097/SCS.0000000000009149 36534028

[14] Sheridan RL Baryza MJ Pessina MA O'Neill KM Cipullo HM Donelan MB. Acute hand burns in children: management and long-term outcome based on a 10-year experience with 698 injured hands. Ann Surg. (1999) 229(4):558–64. 10.1097/00000658-199904000-00016 10203090 PMC1191743

[15] Cho H Ono S Chung KC. Management of scar contractures of the hand-our therapeutic strategy and challenges. J Clin Med. (2024) 13(5):1516. 10.3390/jcm13051516 38592344 PMC10934418

[16] Yang Z Shi X Zhang Y Wang S Lei Z Liu X. Retrospective analysis of factors affecting the efficacy of surgical treatment of the scar. Minerva Chir. (2014) 69(2):83–9. 24504221

[17] Meretsky CR Polychronis A Schiuma AT. A comparative analysis of the advances in scar reduction: techniques, technologies, and efficacy in plastic surgery. Cureus. (2024) 16(8):e66806. 10.7759/cureus.66806 39268283 PMC11392586

[18] Betarbet U Blalock TW. Keloids: a review of etiology, prevention, and treatment. J Clin Aesthet Dermatol. (2020) 13(2):33–43. 32308783 PMC7158916

[19] Yeong EK Chen KW Chan ZH. Risk factors of tissue-expansion failure in burn-scar reconstruction. J Plast Reconstr Aes. (2011) 64(12):1635–40. 10.1016/j.bjps.2011.07.006 21843978

[20] Simpson R Mulay S Nasser A Ibrahim A. 111 Predicting restoration of joint function in the contracted burned hand: the benefit of the soft tissue to skeletal ratio. J Burn Care Res. (2020) 41(Supple1):S74. 10.1093/jbcr/iraa024.114

[21] Abu Alqam R Alshammari AJ Alkhwildi LA Bamatraf MS Khashab RM Al Dwehji AMO. Effectiveness of autologous fat grafting in the treatment of scars: a systematic review and meta-analysis. Aesthet Plast Surg. (2024) 48(19):3945–61. 10.1007/s00266-024-04131-w 39014237

